# Cell segregation and border sharpening by Eph receptor–ephrin-mediated heterotypic repulsion

**DOI:** 10.1098/rsif.2017.0338

**Published:** 2017-07-26

**Authors:** Harriet B. Taylor, Anaïs Khuong, Zhonglin Wu, Qiling Xu, Rosalind Morley, Lauren Gregory, Alexei Poliakov, William R. Taylor, David G. Wilkinson

**Affiliations:** 1 Neural Development Laboratory, The Francis Crick Institute, 1 Midland Road, London NW1 1AT, UK; 2 Computational Cell and Molecular Biology Laboratory, The Francis Crick Institute, 1 Midland Road, London NW1 1AT, UK; 3 Previously at MRC National Institute for Medical Research, The Ridgeway, Mill Hill, London NW7 1AA, UK

**Keywords:** cell segregation, border formation, Eph receptor, adhesion, computer simulation, contact inhibition of locomotion

## Abstract

Eph receptor and ephrin signalling has a major role in cell segregation and border formation, and may act through regulation of cell adhesion, repulsion or tension. To elucidate roles of cell repulsion and adhesion, we combined experiments in cell culture assays with quantitations of cell behaviour which are used in computer simulations. Cells expressing EphB2, or kinase-inactive EphB2 (kiEphB2), segregate and form a sharp border with ephrinB1-expressing cells, and this is disrupted by knockdown of N-cadherin. Measurements of contact inhibition of locomotion reveal that EphB2-, kiEphB2- and ephrinB1-expressing cells have strong heterotypic and weak homotypic repulsion. EphB2 cells have a transient increase in migration after heterotypic activation, which underlies a shift in the EphB2–ephrinB1 border but is not required for segregation or border sharpening. Simulations with the measured values of cell behaviour reveal that heterotypic repulsion can account for cell segregation and border sharpening, and is more efficient than decreased heterotypic adhesion. By suppressing homotypic repulsion, N-cadherin creates a sufficient difference between heterotypic and homotypic repulsion, and enables homotypic cohesion, both of which are required to sharpen borders.

## 1. Introduction

The establishment of organized tissues during development requires that adjacent cell populations with distinct tissue or regional identity do not intermingle with each other. This is reflected by the formation of sharp borders between tissues or regional domains, despite the proliferation and intercalation of cells that can cause intermingling. An important mechanism to stabilize borders is to specifically restrict cells from moving between the adjacent subdivisions. Insights into mechanisms that prevent intermingling have come from experiments in which cells from different tissues are mixed *in vitro*, which for many cell types leads to their segregation. Such segregation occurs during development, on a more local scale, at borders that initially are fuzzy and then sharpen. Three types of mechanisms have been uncovered which can drive segregation and border formation [1–4]. The first is differential adhesion, which has been extensively studied for cadherins that mediate homophilic cell adhesion [5]. At the interface of segregated cell populations, interfacial tension is generated by the imbalance in cohesive forces, which potentially can generate a sharp border if it is sufficient to counter cell motility. A second mechanism is based on increased cortical tension generated by actomyosin contraction [4,6,7]. In classical models, differential adhesion or tension is generated by differences in homotypic adhesion or tension properties of the two cell populations. Recent studies have found that adhesion and tension can also be regulated by interactions of two cell populations such that they are modulated at the heterotypic interface. A third type of mechanism is cell repulsion, in which contact triggers a rapid local retraction of cell processes. For migratory cells, such repulsion is a component of the contact inhibition of locomotion, in which contact leads to a change in the direction of movement [8,9].

Eph receptor tyrosine kinase and ephrin signalling has a major role in boundary formation [2,10,11]. Eph receptors and ephrins are cell surface bound molecules that upon interaction can activate signal transduction in both cells [12,13]. Commonly, expression of Eph receptors and ephrins that have high affinity is complementary, and strong activation thus occurs at the interface [14]. Such activation has been implicated in preventing intermingling between tissues or regional domains [15–19]. Further evidence has come from cell culture assays in which Eph receptor and ephrin-expressing cells segregate from each other and their intermingling is inhibited [20–22]. Eph–ephrin signalling has been shown to regulate mechanisms at heterotypic contacts that can potentially drive cell segregation and border sharpening [10,11]: by decreasing cadherin-mediated adhesion [17,23], by cell repulsion [22,24] and by increasing cortical tension [25]. However, the relative importance of these mechanisms, and whether they are sufficient to account for cell segregation and border sharpening remain unclear. This can be addressed by quantitating cell behaviours and using these in computer simulations.

Computer simulations with values measured from cells using the Cellular Potts model support that differential adhesion or tension can drive segregation [26,27] and border sharpening [7], though they have yet to be applied to Eph–ephrin cell segregation. These simulations predict cell rearrangements in epithelia based on minimization of free energy, but do not include cell migration as a mechanism. Simulations of repulsive interactions of migratory cells suggest that Eph cell repulsion could drive segregation [28], but did not use parameters from measurements of cell behaviour or examine whether cell repulsion is sufficient for border sharpening.

We established assays in which HEK293 cells with stable expression of EphB2 and ephrinB1 segregate from each other [22,29], and developed an agent-based simulation of migratory cells [30,31]. Here, we use this assay system to determine whether cell repulsion and/or decreased heterotypic compared with homotypic adhesion underlies cell segregation and border sharpening. We test the effect of altering EphB2 signalling and of N-cadherin knockdown, measure the migratory and adhesive behaviour of cells, and use these measurements in simulations. We show that heterotypic repulsion, but not decreased heterotypic adhesion, is sufficient to segregate cells and sharpen borders. N-cadherin contributes to cell segregation and border sharpening by suppressing excessive homotypic repulsion.

## 2. Results

### 2.1. EphB2–ephrinB1-mediated segregation and border sharpening

**Figure 1.**
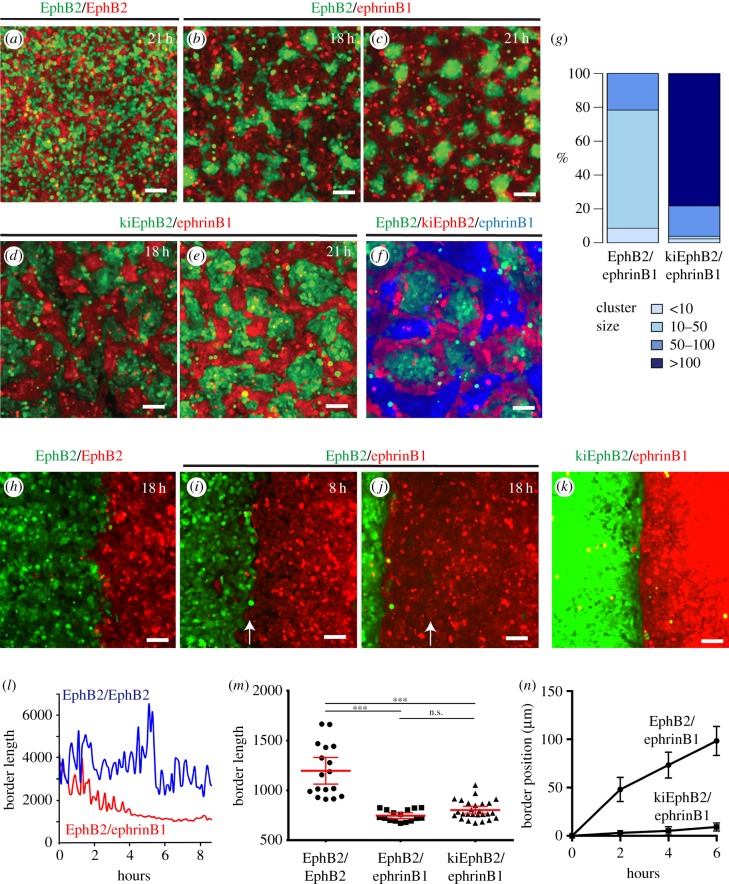
Cell segregation and boundary assays. (*a*–*f*) Segregation assays for (*a*) EphB2/EphB2; (*b*,*c*) EphB2/ephrinB1; (*d*,*e*) kiEphB2/ephrinB1; (*f*) EphB2/kiEphB2/ephrinB1. (*g*) Quantitation of the number of cells in EphB2 cell clusters; (*h*–*k*) boundary assays for (*h*) EphB2/EphB2 at 18 h; (*i*,*j*) EphB2/ephrinB1 at 8 and 18 h after barrier removal. Arrows indicate the position where the border first forms, which then moves towards the EphB2 cell population; (*k*) kiEphB2/ephrinB1. (*l*) Quantitation of border length (pixels) from when the cell populations meet. (*m*) Quantitation of border length (pixels) after greater than 6 h interaction. ***p < 0.0001; n.s., not significant. (*n*) Quantitation of position of the border from when the two cell populations meet. The colour of the different cells is indicated by the figure text; scale bar, 100 µm.

**Figure 2.**
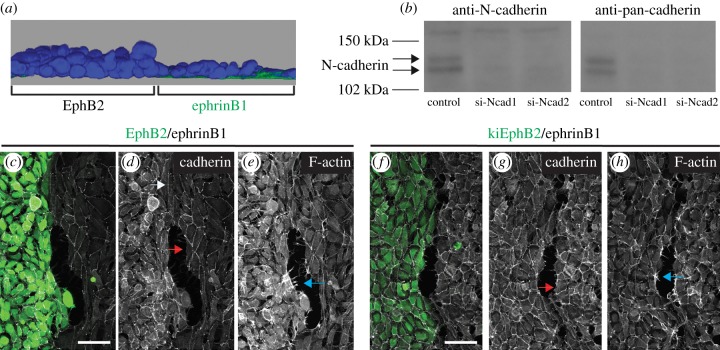
N-cadherin and F-actin distribution. (*a*) z-stack images from an EphB2/ephrinB1 boundary assay reconstructed to visualize from the side. EphB2 cells pile on top of each other at the border. (*b*) Western blot analysis of HEK293 cells following treatment with control siRNA, Dharmacon Smartpool N-cadherin siRNA (si-Ncad1) or Ambion N-cadherin siRNA (si-Ncad2), probed with N-cadherin or pan-cadherin antibody. The absence of other proteins detected by pan-cadherin antibody suggests that other classical cadherin family members have low or no expression. (*c*–*h*) Detection of N-cadherin by immunostaining and F-actin with phalloidin in EphB2/ephrinB1 and kiEphB2/ephrinB1 boundary assays. Where cells are in contact at the heterotypic interface, N-cadherin staining (white arrows) is similar in level as at homotypic interfaces, but lower where cells are not in contact (red arrows). Actin-rich fibres extend into gaps between cells at the heterotypic interface (blue arrows). Scale bar, 50 µm.

In segregation assays, HEK293 cells with stable expression of EphB2 or ephrinB1 (for brevity, termed EphB2 and ephrinB1 cells) are labelled with fluorescent dyes, and are mixed and plated at sub-confluent density [22]. The two populations segregate to form EphB2 cell clusters surrounded by ephrinB1 cells (figure 1*a*,*b*), and the EphB2 cell clusters subsequently become compacted (figure 1*c*). In experiments to test potential roles of kinase-independent signalling, we generated HEK293 cells expressing a kinase-inactive mutant (K661R) of EphB2, termed kiEphB2 cells. We found that kiEphB2 and ephrinB1 cells segregate (figures 1*d*,*e* and 2*e*,*k*), but rather than forming islands, kiEphB2 cells are interconnected and thus have a greater cluster size than EphB2 cells (figure 1*g*). Also, unlike EphB2 cells, kiEphB2 cell clusters do not undergo subsequent compaction. To further test the behaviour of kiEphB2 cells, we carried out segregation assays in which EphB2, kiEphB2 and ephrinB1 cells are mixed. kiEphB2 cells selectively segregate to the interface with ephrinB1 cells, with EphB2 cells preferentially located at the centre of EphB2/kiEphB2 cell clusters (figure 1*f*): kiEphB2 cells comprise 44% (560/1267) of the total EphB2 + kiEphB2 cells, but 84% (118/140) of the cells at the interface with ephrinB1 cells. This pattern is consistent with kiEphB2 cells having a weaker heterotypic response than EphB2 cells, which nevertheless drives segregation from ephrinB1 cells. Based on these findings, it was informative to analyse kiEphB2 cells and EphB2 cells in subsequent studies.

As border sharpness is difficult to quantitate due to the variable shape of cell clusters, we used an assay in which cells are plated on each side of a barrier, grown to confluence and the barrier removed. The cells invade the gap and form an initially ragged border. In EphB2–EphB2 cell controls, the border remains ragged (figure 1*h*), whereas the EphB2–ephrinB1 cell border becomes sharp (figure 1*i*,*j*). To quantitate sharpness, we measured the length of the border, and found that sharpening occurs over a 3–4 h period following contact between EphB2 and ephrinB1 cells (figure 1*l*). Concurrent with sharpening, the border shifts in the direction of the EphB2 cell population (figure 1*i*,*j*,*n*) and EphB2 cells pile up (figure 2*a*). By contrast, kiEphB2 and ephrinB1 cells have only a small movement of the border in boundary assays (figure 1*n*). Remarkably, the kiEphB2/ephrinB1 border is as sharp as that for EphB2 and ephrinB1 cells (figure 1*k*,*m*).

### 2.2. N-cadherin and cortical actin in EphB2 and ephrinB1 cells

To address potential roles of cadherin-mediated adhesion and/or assembly of actomyosin cables, we analysed whether there is modulation of cadherin levels and actin cable assembly at the heterotypic interface. We immunostained for N-cadherin, which is the predominant classical cadherin expressed by HEK293 cells (figure 2*b*), and stained for F-actin with phalloidin. We found higher levels of N-cadherin and F-actin in EphB2 cells compared with ephrinB1 cells (figure 2*c*–*e*), but not in kiEphB2/ephrinB1 assays (figure 2*f*–*h*). These findings suggest that the increased cadherin and actin staining is secondary to the compaction and piling up of EphB2 cells (figure 2*a*), which does not occur for kiEphB2 cells. Where EphB2 or kiEphB2 cells are in contact with ephrinB1 cells, N-cadherin is at a similar level as at homotypic interfaces (see electronic supplementary material, figure S1*a*), but is at lower levels at the cell surface where cells are not in contact (figure 2*d*,*g*; electronic supplementary material, figure S1*c*). F-actin is not specifically elevated where EphB2 or kiEphB2 and ephrinB1 cells are in contact (see electronic supplementary material, figure S1*b*), but is seen associated with fibres that protrude into the gaps between cells (figure 2*e*,*h*; electronic supplementary material, figure S1*d*). These findings are consistent with cell repulsion following Eph–ephrin signalling that leads to actin-rich retraction fibres, and removal of N-cadherin at the free cell surface due to loss of trans-interactions that stabilize cadherins [32]. The presence or absence of a gap and retraction fibres at the heterotypic interface may reflect different phases in a cycle of adhesion and repulsion, as found in other contexts [15].

### 2.3. Heterotypic and homotypic cell repulsion

**Figure 3.**
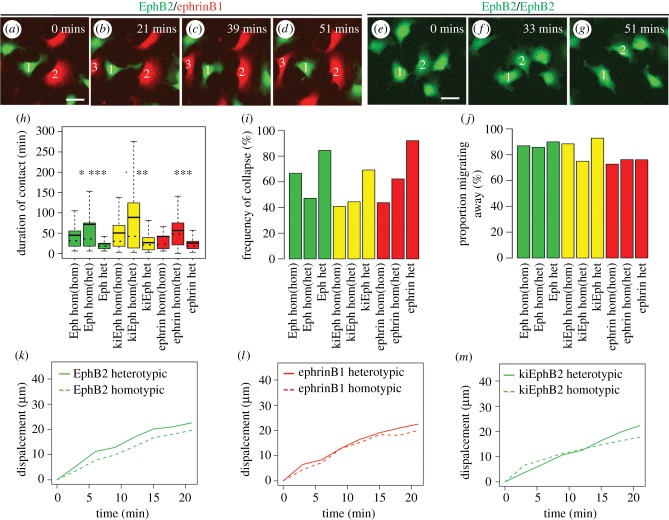
Homotypic and heterotypic cell repulsion. Time lapse movies were captured of labelled cells in homotypic or heterotypic culture. (*a*–*d*) Snapshots of electronic supplementary material, movie S2 in which heterotypic repulsion occurs between EphB2 cell 1 and ephrinB1 cells 2 and 3. (*e*–*g*) Snapshots of electronic supplementary material, movie S3 in which homotypic repulsion occurs between EphB2 cells 1 and 2. Manual tracking was used to calculate the duration of contact (*h*), frequency of collapse response after contact (*i*) and proportion of cells that migrate away from the interacting cell after contact (*j*). het, heterotypic repulsion; hom(hom), homotypic repulsion in homotypic culture; hom(het), homotypic repulsion in heterotypic culture. (*k*–*m*) Quantitation of average displacement of EphB2 cells (*k*), ephrinB1 cells (*l*) or kiEphB2 cells (*m*) after homotypic or heterotypic repulsion. ***p < 0.001, **p < 0.01, *p < 0.05. Scale bar, 20 µm.

The above findings argue against segregation being driven by a decrease in N-cadherin or by assembly of actin cables. We therefore wondered whether cell segregation and border sharpening is driven by cell repulsion. Indeed, even at high density, EphB2 and ephrinB1 cells were motile and had frequent repulsion responses, manifested by rapid retraction of cell processes and movement away from the other cell (see electronic supplementary material, movie S1). To measure responses to individual contacts, we plated cells at low density, such that contact inhibition of locomotion can be measured. We refer to the cell response as repulsion, because this term also applies to the behaviour in confluent culture when cells are constrained from migrating away from each other. Heterotypic repulsion was found to occur between EphB2 and ephrinB1 cells (figure 3*a*–*d*; electronic supplementary material, movie S2) and between kiEphB2 and ephrinB1 cells (see electronic supplementary material, movie S3). Repulsion also occurs following homotypic interactions of EphB2, kiEphB2 and ephrinB1 cells (figure 3*e*–*g*; electronic supplementary material, movies S4–S6).

To quantitate the relative strength of cell responses, we tracked approximately 46 cells in movies for each experimental condition. Each of the cells had 27 ± 1.7 cell contacts during the movie, from which we measured four parameters: duration of contact (figure 3*h*); frequency with which contact leads to a repulsion response (figure 3*i*); the direction of cell migration after contact (figure 3*j*; electronic supplementary material, figure S2); and the displacement of cells following disengagement (figure 3*k*–*m*). At a mechanistic level, the duration of cell–cell contact probably reflects a combination of cell adhesion, tension and the retraction of cell processes during repulsion. We found that, for EphB2 cells, interactions with ephrinB1 cells have a mean duration of 25 min, with 84% of collisions leading to a collapse response. Homotypic repulsion of EphB2 cells was less strong, and weaker in EphB2/ephrinB1 compared with EphB2 cell culture: there was a mean contact duration of 72 min versus 45 min and collapse response after 47% versus 67% of collisions, respectively. These findings suggest that heterotypic interactions desensitize EphB2 cells to homotypic repulsion. kiEphB2 cells had a lower frequency of collapse following heterotypic interactions (70%), but otherwise similar behaviour to EphB2 cells, including decreased homotypic repulsion in heterotypic cell culture (figure 3*h*,*i*). Likewise, ephrinB1 cells had strong heterotypic and less strong homotypic repulsion, which decreased in EphB2/ephrinB1 compared with ephrinB1 cell cultures (figure 3*h*). Upon disengaging, EphB2 and ephrinB1 cells usually migrate away from the interacting partner (figure 3*j*), in a similar proportion for homotypic and heterotypic interactions, but higher for EphB2 cells (86–90%) than for ephrinB1 cells (73–76%). Analysis of the displacement of cells following collisions revealed that, during heterotypic repulsion, EphB2 cells have an increase in migration speed for approximately 6 min, which does not occur during homotypic repulsion (figure 3*k*). An increase in migration speed also occurs following heterotypic activation of ephrinB1 cells, but it is more transient than for EphB2 cells (figure 3*l*). By contrast, heterotypic activation of kiEphB2 cells does not lead to a burst in migration (figure 3*m*). These findings reveal that EphB2, kiEphB2 and ephrinB1 cells each have strong heterotypic and less strong homotypic repulsion. The greater displacement of EphB2 cells than ephrinB1 cells following heterotypic interaction may account for the compaction of EphB2 cell clusters and border shifting. This sustained migration response is not essential for cell segregation or border sharpening as it does not occur upon heterotypic activation of kiEphB2 cells, which segregate and form a sharp border with ephrinB1 cells.

### 2.4. Simulations of cell segregation and border sharpening

We further developed agent-based computer simulations [30] to test whether the measured cell behaviours can account for cell segregation and border sharpening. The cell surface is represented as eight linked nodes, each of which can be assigned migration and adhesion properties. To simulate migration, one node is designated as leading the direction of migration. In repulsion, when cells collide, a node distal from the site of cell–cell contact is assigned a high probability of becoming a leader, and thus the cells move away. In adhesion, nodes of different cells that come into contact become linked, and the probability of unlinking determines the duration of contact. Simulations of segregation were carried out with 400 green (EphB2 cell parameters) and 400 red (ephrinB1 cell parameters) cells that are intermingled at the start (see electronic supplementary material, figure S3). In boundary simulations, each of the two cell populations occupies one-half of the field, with a fuzzy border at the beginning (see electronic supplementary material, figure S4).

**Figure 4.**
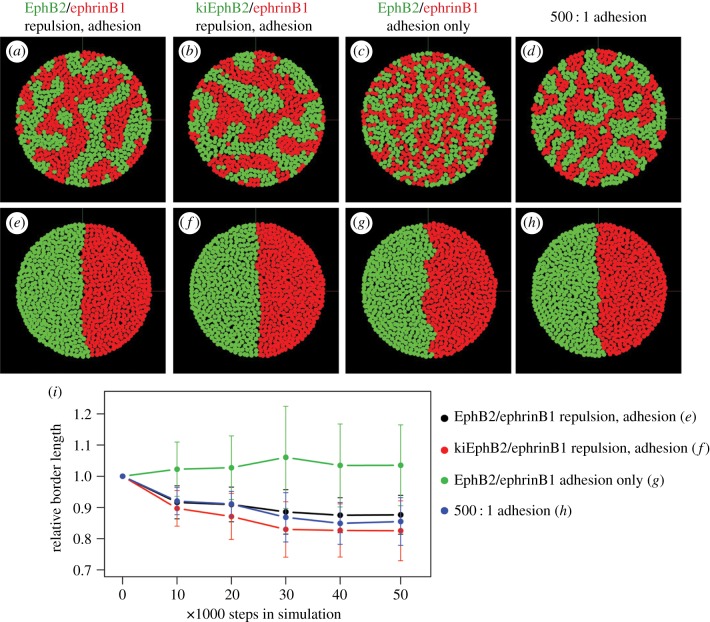
Simulations of cell adhesion and repulsion. Simulations of cell segregation (*a*–*d*) and border sharpening (*e*–*h*) carried out for 50 000 steps. ‘Adhesion’ refers to simulations of contact duration, which for cells is determined by the strength of adhesion, tension and repulsion. ‘Repulsion’ refers to simulations in which cells move away directionally after contact. The simulations were carried out with the following parameters. (*a*,*e*) Directional repulsion and adhesion values of EphB2 and ephrinB1 cells. (*b*,*f*) Directional repulsion and adhesion values of kiEphB2 and ephrinB1 cells. (*c*,*g*) Adhesion values of EphB2 and ephrinB1 cells with random migration. (*d*,*h*) 500 : 1 ratio of homotypic: heterotypic contact duration with random migration. (*i*) Time course of average border length calculated from 10 simulations of border sharpening, with standard error indicated. Border sharpness for adhesion with random migration (*g*) was significantly different from the other conditions (*p*-value 2 × 10^−5^).

We carried out simulations of segregation using the homotypic and heterotypic contact duration and repulsion values measured from movies of cell assays (end points in figure 4*a*–*e*; time courses in electronic supplementary material, figure S3). We found that the values measured for EphB2/ephrinB1 (figure 4*a*) and for kiEphB2/ephrinB1 interactions (figure 4*b*) both lead to strong segregation. Unlike cell assays, in simulations EphB2 cells do not form islands. This may reflect that the two-dimensional simulations do not include the compaction of clusters or piling up of cells seen in cell culture. The shorter duration of heterotypic contact may reflect the strength of repulsion which leads to cell–cell disengagement. However, an alternative model is that Eph-ephrin interactions drive segregation by decreasing adhesion at heterotypic contacts with no change in directional migration. To test this, we carried out simulations in which the threefold shorter duration of heterotypic compared with homotypic contact is assumed to reflect only cell adhesion, with random cell migration after contact. We found that these values of homotypic and heterotypic contact duration drive only a low amount of segregation (figure 4*c*). Greater segregation occurs if the ratio of homotypic/heterotypic contact duration is increased, but even with a 500-fold ratio (figure 4*d*) is less extensive than achieved by differential repulsion. Thus, although decreased heterotypic compared with homotypic adhesion can drive segregation, it does not account for the extent of segregation of EphB2 and ephrinB1 cells.

To test whether repulsion and/or decreased heterotypic adhesion could account for border sharpening, we carried out boundary assay simulations with the same parameters described above (figure 4*e*–*h*). Border sharpness was calculated by determining the relative border length (figure 4*i*; time courses in electronic supplementary material, figure S4). We found that the measured contact duration and repulsion values for EphB2/ephrinB1 (figure 4*e*,*i*) and kiEphB2/ephrinB1 (figure 4*f*,*i*) lead to border sharpening. By contrast, border sharpening does not occur in simulations of homotypic and heterotypic adhesion using the measured values of contact duration (figure 4*g*,*i*). Border sharpening does occur with a 500-fold difference in contact duration (figure 4*h*,*i*). In summary, simulations show that heterotypic repulsion but not decreased heterotypic adhesion with the values measured for EphB2 and ephrinB1 cells is sufficient to drive segregation and border sharpening.

### 2.5. N-cadherin contributes to segregation and border sharpening

**Figure 5.**
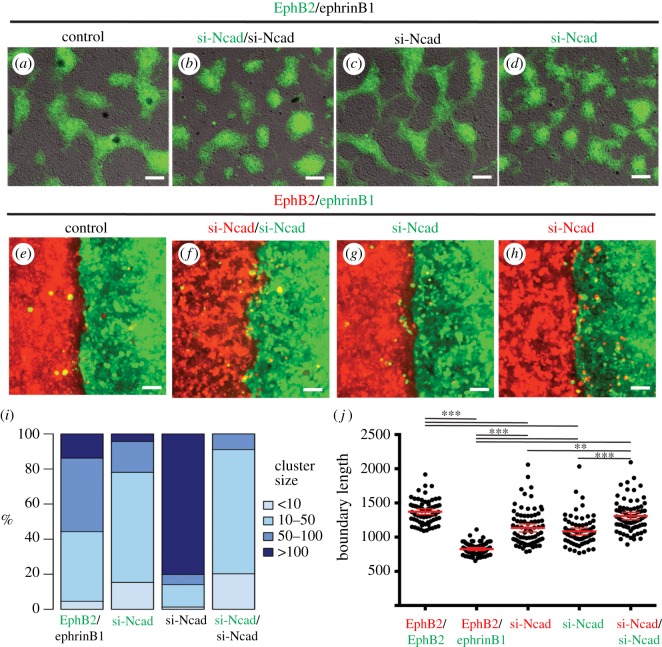
Effect of N-cadherin knockdown on segregation and border sharpness. N-cadherin knockdown (si-Ncad) was carried out in segregation (*a*–*d*) and boundary assays (*e*–*h*) with EphB2 and ephrinB1 cells. The colour of the si-Ncad label indicates which cell population(s) the knockdown has been carried out in. The size of EphB2 cell clusters in segregation assays is quantitated in (*i*), and border sharpness in boundary assays is quantitated in (*j*). ***p < 0.0001, **p < 0.001. Scale bar, 100 µm.

In epithelial cell lines, E-cadherin knockdown disrupts cell segregation driven by Eph–ephrin signalling [21], so we wondered whether there is an analogous requirement for N-cadherin in EphB2 and ephrinB1 cells. We found that segregation still occurs following N-cadherin knockdown (figure 5*a*,*b*), but EphB2 cell clusters are smaller than in control EphB2/ephrinB1 assays (figure 5*i*). We tested whether N-cadherin is required selectively in EphB2 cells or ephrinB1 cells by knockdown in one or the other cell population. Segregation occurs in both situations, but with a striking difference in cell organization. Following N-cadherin knockdown in ephrinB1 cells, nearly EphB2 cells are in contact through narrow bridges between aggregates, increasing cluster size as measured by the number of contiguous EphB2 cells (figure 5*c*,*i*). Conversely, EphB2 cell clusters are smaller and less interconnected following N-cadherin knockdown only in EphB2 cells (figure 5*d*,*i*). These differences in cell organization suggest that N-cadherin contributes to but is not essential for segregation, and promotes homotypic contact within the segregated cells. We found that knockdown of N-cadherin in both EphB2 and ephrinB1 cells in boundary assays leads to a major disruption in border sharpening (figure 5*e*,*f*,*j*). We tested whether N-cadherin is required selectively in EphB2 or ephrinB1 cells and found that knockdown of N-cadherin in either cell population leads to a similar decrease in border sharpness, with a greater decrease following knockdown in both cell populations (figure 5*g*,*h*,*j*). N-cadherin is thus required both in EphB2 and ephrinB1 cells to enable border sharpening.

### 2.6. N-cadherin suppresses homotypic repulsion

**Figure 6.**
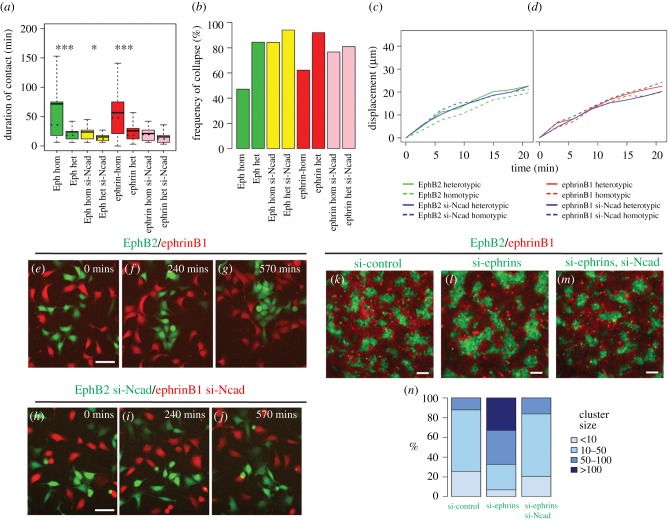
Effect of N-cadherin and endogenous ephrin knockdown on cell repulsion and segregation. (*a*–*j*) Time lapse movies were made of EphB2 and ephrinB1 cells in segregation assays with or without N-cadherin knockdown. Cell tracking was used to calculate the duration of contact (*a*), frequency of collapse response after contact (*b*), and displacement after repulsion (*c*,*d*). hom, homotypic; het, heterotypic; si-Ncad, N-cadherin knockdown. Data for assays without N-cadherin knockdown (figure 3) are included for comparison. (*e*–*g*) In a control assay (see electronic supplementary material, movie S7), heterotypic repulsion leads to formation of an EphB2 cell cluster which is maintained. (*h*–*j*) Following N-cadherin knockdown (see electronic supplementary material, movie S8), the EphB2 cell cluster that forms is repeatedly broken up by homotypic repulsion. (*k*–*m*) Cell segregation assays were carried out following knockdown with control (*k*), ephrinB1 and ephrinB2 (*l*) or ephrinB1, ephrinB2 and N-cadherin (*m*) siRNA in EphB2 cells. (*n*) Quantitation of cluster size. ***p < 0.001, *p < 0.05. Scale bars, 50 µm (*e*–*j*), 100 µm (*k*–*m*).

We wondered whether, rather than reflecting a requirement for a difference between heterotypic and homotypic adhesion [21], the disruption of Eph–ephrin cell segregation by cadherin knockdown is due to altered repulsion. We quantitated cell responses in low-density cultures and found that N-cadherin knockdown leads to a major change in homotypic responses of EphB2 cells: the duration of contact decreases to 24 min, the frequency of collapse responses increases to 84% and there is a burst of migration during repulsion (figure 6*a*–*c*). These cell responses are similar to heterotypic repulsion of EphB2 cells in the presence of N-cadherin. N-cadherin knockdown also decreases contact duration (15 min) and increases the collapse frequency (94%) of heterotypic EphB2 cell interactions, but does not further increase cell displacement during repulsion (figure 6*a*–*c*). Likewise for ephrinB1 cells, N-cadherin knockdown decreases the contact duration and increases the collapse frequency of both homotypic (21 min, 77%) and heterotypic (15 min, 81%) interactions (figure 6*a*,*b*). N-cadherin knockdown has little effect on the displacement of ephrinB1 cells during homotypic or heterotypic repulsion (figure 6*d*).

Based on these findings, we wondered whether increased homotypic repulsion may account for decreased segregation following N-cadherin knockdown. We took advantage of finding that EphB2 cell clusters start to form at low density in segregation assays. In movies of assays with no knockdown of N-cadherin we observed that heterotypic repulsion pushes EphB2 cells together into an increasingly large cluster, and despite homotypic repulsion, the EphB2 cells remain in close contact (figure 6*e*–*g*; electronic supplementary material, movie S7). By contrast, following N-cadherin knockdown, EphB2 cells form clusters, but these do not grow as they are broken up by frequent homotypic repulsion (figure 6*h*–*j*; electronic supplementary material, movie S8). These observations suggest that suppression of homotypic repulsion by N-cadherin enables cohesion that stabilizes cell clusters during segregation.

### 2.7. Overlapping Eph–ephrin expression contributes to homotypic repulsion

Although Eph receptors and ephrins with high affinity have complementary expression, there are also overlaps of lower-affinity partners that can mediate homotypic repulsion [16]. We therefore measured by qPCR the relative expression levels of endogenous EphB receptors and ephrinBs in HEK293 cells (table 1). Taking into account binding affinities [33], these findings predict that ephrinB1 activates EphB2, with lower contributions from ephrinB2 and ephrinB3, and that EphB2 activates ephrinB1, with a lower contribution from EphB1. We carried out knockdowns to test whether these endogenous ephrins and Eph receptors underlie homotypic repulsion.

**Table 1.** Relative expression of EphB receptor and ephrinB mRNA in HEK293 cells.

	relative expression level normalized to ephrinB1
EphB1	19.5
EphB2	57.7
EphB3	0.82
EphB4	75.4
EphB6	11.2
ephrinB1	100
ephrinB2	34.5
ephrinB3	27.6

We found in segregation assays that knockdown of ephrinB1 plus ephrinB2 in EphB2 cells increases the size of EphB2 cell clusters compared with control knockdowns, and that this increase is abrogated by simultaneous knockdown of N-cadherin (figure 6*k*–*m*, quantitation in figure 6*n*). These findings support that ephrinB1 and ephrinB2 contribute to homotypic repulsion of EphB2 cells that is opposed by N-cadherin. In experiments to test roles of Eph receptors in ephrinB1 cells, knockdown of EphB1 has no apparent effect, whereas after EphB2 knockdown, the cells have a round shape with greatly decreased motility (data not shown). This disruption of cell motility precluded analysis of whether EphB receptors contribute to homotypic repulsion of ephrinB1 cells.

### 2.8. Simulations of cell behaviour following knockdown of N-cadherin

**Figure 7.**
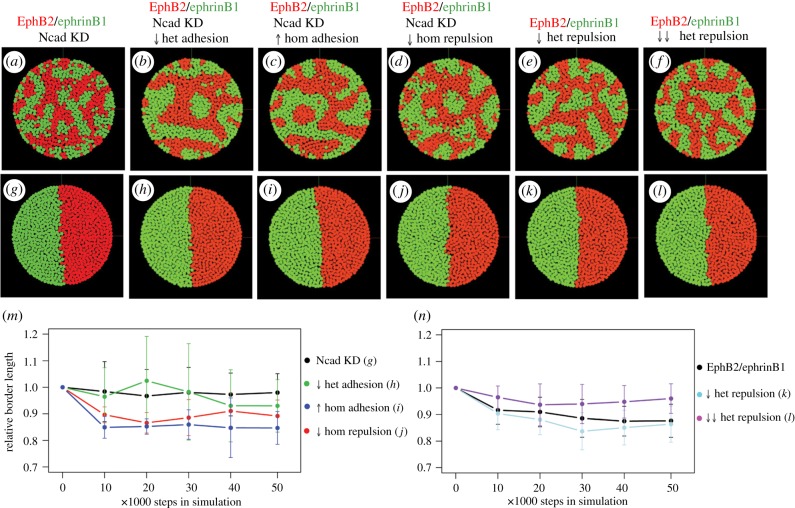
Simulations of N-cadherin knockdown. Simulations of cell segregation (*a*–*f*) and border sharpening (*g*–*l*) carried out for 50 000 steps with the following parameters. (*a*,*g*) Directional repulsion and adhesion (contact duration) values of EphB2 and ephrinB1 cells following N-cadherin knockdown; (*b*,*h*) N-cadherin knockdown values but decreasing the duration of heterotypic contact from 15 to 8 min; (*c*,*i*) N-cadherin knockdown values but increasing the duration of homotypic contact from 24 to 45 min; (*d*,*j*) N-cadherin knockdown values but decreasing the frequency of homotypic repulsion from 0.47 to 0.25 (EphB2) and from 0.62 to 0.3 (ephrinB1); (*e*,*k*) EphB2 and ephrinB1 control values (figure 4*a*,*f*) but reducing the frequency of heterotypic repulsion from 0.84 to 0.53 (EphB2) and from 0.92 to 0.65 (ephrinB1); (*f*,*l*) EphB2 and ephrinB1 control values but reducing the frequency of heterotypic repulsion to 0.27 (EphB2) and 0.36 (ephrinB1). (*m*) Time course of average border length in simulations *g*–*j*, calculated from 10 runs. Border sharpness significantly increased following decreased homotypic repulsion (*j*; *p*-value 0.032) and increased homotypic adhesion (*i*; *p*-value 0.016) compared with N-cadherin knockdown parameters. (*n*) Time course of average border length in simulations *k*, *l* and control EphB2/ ephrinB1, calculated from 10 runs. Border sharpness significantly decreased following the greater decrease in heterotypic repulsion frequency (*l*; *p*-value 0.036) compared with EphB2/ephrinB1 control parameters.

N-cadherin knockdown increases the frequency of homotypic and heterotypic collapse responses and decreases the duration of cell–cell contact. We carried out simulations to test whether these changes could account for the observed decrease in cell segregation and border sharpening. We found that simulations with the contact duration and repulsion values following N-cadherin knockdown had decreased segregation and disrupted border sharpening (figure 7*a*,*g*,*m*) compared with controls (figure 4*a*,*f*,*k*). This could occur because of a destabilizing effect of the increase in homotypic repulsion which decreases the duration of homotypic contact (see electronic supplementary material, movies S7 and S8). Alternatively, or in addition, segregation and border sharpening may require a large enough difference between homotypic and heterotypic cell repulsion, which is diminished by N-cadherin knockdown. As a measure of relative repulsion strength we calculated the repulsion frequency per minute = (collapse frequency per contact)/(duration of contact). From this, we calculated the ratio of heterotypic/homotypic repulsion = (heterotypic repulsion frequency)/(homotypic repulsion frequency). For EphB2 cells and ephrinB1 cells, the repulsion ratio is 5.36 and 3.25, respectively. Following N-cadherin knockdown, the repulsion ratio is reduced to 1.79 and 1.47, respectively.

We carried out simulations to test whether segregation and border sharpening are influenced by the repulsion ratio and/or the duration of homotypic interactions. Starting with N-cadherin knockdown parameters, we decreased the duration of heterotypic contact, which increases the repulsion ratio to 3.4, and found that with these conditions there was an increase in segregation but the border still failed to sharpen (figure 7*b*,*h*,*m*). By contrast, border sharpening as well as increased segregation occurred when homotypic contact duration was lengthened from 24 to 45 min (figure 7*c*,*i*,*m*), which creates the same repulsion ratio of 3.4. Increased segregation and border sharpening also occurred, but to a lesser extent, when the homotypic repulsion frequency was decreased while keeping the duration of homotypic contact constant, to create a repulsion ratio of 6 (figure 7*d*,*j*,*m*). This suggests that border sharpening is promoted by increasing the duration of homotypic contact, and that the shorter duration of contact following N-cadherin knockdown can only be partly compensated for by increasing the ratio of heterotypic/homotypic repulsion.

The above simulations did not cleanly dissect whether a high ratio of heterotypic/homotypic repulsion is required. To test this, we used control EphB2/ephrinB1 parameters, keeping homotypic contact duration and repulsion fixed and decreasing the frequency of heterotypic repulsion. We found that segregation and border sharpening still occurred after reducing the repulsion ratio from control values of 5.36 and 3.25 (EphB2 and ephrinB1, respectively) to 3.38 and 2.3 (figure 7*e*,*k*,*n*), but was disrupted when this was further reduced to 1.72 and 1.27 (figure 7*f*,*l*,*n*), similar to the values following N-cadherin knockdown. Taken together, these simulations suggest that the increase in homotypic repulsion following N-cadherin knockdown disrupts border sharpening both by decreasing the duration of homotypic cell contacts, and by decreasing the ratio of heterotypic/homotypic repulsion.

## 3. Discussion

We set out to identify mechanisms that drive Eph–ephrin-mediated segregation and border sharpening, and the relationship with cadherin function. We quantitated EphB2 and ephrinB1 cell behaviour and carried out simulations to test whether specific behaviours can in principle contribute, or be sufficient for segregation and border sharpening. Our findings reveal that heterotypic repulsion can drive cell segregation and border sharpening, and that there is a critical requirement to suppress homotypic repulsion.

### 3.1. Segregation by heterotypic repulsion

Our findings strongly support that, for HEK293 cells, which are motile and have transient adhesive interactions, segregation is primarily driven by heterotypic repulsion. In movies of low-density cultures, collision of EphB2 and ephrinB1 cells leads to contact inhibition of locomotion, in which there is localized collapse of cell processes of one or both cells, followed by migration away from the heterotypic partner. Where EphB2 and ephrinB1 cells are in contact in confluent culture, there are similar levels of N-cadherin as at homotypic contacts, and we did not observe an increased assembly of actin cables. Less N-cadherin was seen at sites where there are gaps between EphB2 and ephrinB1 cells, and actin-rich retraction fibres were often seen in these gaps. The gaps at the heterotypic interface are probably due to repulsion, which secondarily leads to loss of N-cadherin at the cell surface. That heterotypic repulsion underlies segregation is supported by movies at low density, in which repeated repulsion by ephrinB1 cells is seen to push EphB2 cells together to form clusters. Interestingly, the duration of EphB2–EphB2 cell contact is greater in heterotypic compared with homotypic culture, thus increasing both the stability of EphB2 cell clusters and the heterotypic/homotypic repulsion ratio. Likewise, ephrinB1 cells have an increased duration of homotypic contact in heterotypic compared with homotypic culture. This change in behaviour is suggestive of desensitization of repulsion by EphB2–ephrinB1 interactions, which most strongly affects homotypic responses.

Our experimental findings are supported by simulations which show that heterotypic repulsion is sufficient to drive cell segregation. By contrast, only weak segregation occurred in simulations of differences in homotypic and heterotypic adhesion based on the measured durations of contact. This may reflect that the simulations incorporate the high motility of HEK293 cells, and the difference in adhesion is insufficient to overcome the disruptive effect of cell migration. Indeed, segregation does occur in simulations with a larger difference between homotypic and heterotypic contact duration. Our findings therefore support that differential repulsion is the main mechanism that drives segregation of EphB2 and ephrinB1 cells. The key behaviour that distinguishes differential repulsion, and increases the efficiency of segregation, is the migration of cells away from each other rather than random migration after contact.

It is important to note that the cell behaviours that were measured and used in the simulations reflect the net effect of multiple cellular mechanisms. In particular, the duration of cell–cell contact is not directly related to the amount of cell adhesion, but rather will be influenced by the relative strength of adhesion and tension, and by the collapse of cell processes during repulsion. The shorter duration of cell contacts following N-cadherin knockdown probably reflects increased tension and repulsion frequency as well as a decrease in adhesion.

Recent work has presented evidence for an alternative mechanism, in which the segregation of EphB2 and ephrinB1 cells is driven by a difference in cortical tension at the EphB2/ephrinB1 interface [34]. This study proposed that migration following repulsion does not drive segregation, because EphB2 cell clustering was not observed in low-density culture. By contrast, we show that EphB2 cell clusters start to form at low density, prior to sustained contact with ephrinB1 cells, and cell repulsion responses occur both at low and high density. Evidence for a tension-based mechanism came from detection of a higher amount of F-actin at the EphB2–ephrinB1 cell interface [34]. The current study too finds an increased level of F-actin staining, but reveals that accumulation of actin is associated with retraction fibres in gaps between cells at the EphB2–ephrinB1 interface, rather than formation of cortical actin cables. As tension requires cell–cell adhesion [35], it can underlie segregation in epithelial tissues, whereas cell repulsion occurs in mesenchymal cells which have transient adhesive interactions. Indeed, it is likely that the dominant mechanism for cell segregation depends upon the environment. In the assays used here, HEK293 cells are migrating on extracellular matrix and, consequently, repulsion is the main driver of cell segregation. Differential adhesion or tension is likely to be the dominant mechanism in epithelial tissues and other contexts in which cells are less migratory.

### 3.2. Border sharpening by heterotypic repulsion

The results of simulations suggest that the ratio of heterotypic/homotypic repulsion measured for EphB2 and ephrinB1 cells (5.36 and 3.25, respectively) is sufficient to drive border sharpening. Sharpening still occurs with a lower repulsion ratio (3.38 and 2.3) but not when further reduced to 1.72 and 1.27, presumably reflecting the amount of differential repulsion required to overcome the disruptive effect of cell motility. Simulations reveal that decreased heterotypic compared with homotypic adhesion with random migration is not able to drive border sharpening when set at the ratio of homotypic/heterotypic contact duration measured for EphB2 (3.0) and ephrinB1 cells (2.2). Border sharpening does occur when the relative duration of cell contact is set at a much higher ratio (500-fold). This suggests that migration of cells away from each other after heterotypic interaction is a more efficient mechanism than differences in homotypic and heterotypic adhesion for border sharpening. Directional repulsion will also inhibit cells from invading heterotypic territory.

In boundary assays, the border shifts in the direction of EphB2 cells, and in segregation assays this directional movement accounts for the compaction of EphB2 cell clusters surrounded by ephrinB1 cells. Similar to these findings, recent work has shown that up-regulation of EphA2 in Ras-transformed epithelial cells underlies the compaction and extrusion of the EphA2-expressing cells [36]. This extrusion is due to repulsion of EphA2-expressing cells, which underlies shifting of the border in assays in which Ras-transformed and non-transformed MDCK cells are confronted [36]. Our findings can be explained by asymmetric cell migration, in which EphB2 cells have a more sustained migration response than ephrinB1 cells during heterotypic repulsion. In support of this, border shifting is reduced and compaction of clusters does not occur in assays with kiEphB2 cells, which are repelled but do not have a burst of increased migration after heterotypic interaction. As kiEphB2 cells form a sharp border with ephrinB1 cells, the sustained migration response of EphB2 cells is not required for border sharpening. The stronger migration response of EphB2 cells can explain why when EphB2, kiEphB2 and ephrinB1 cells are mixed, kiEphB2 cells preferentially segregate to the interface with ephrinB1 cells and EphB2 cells to the centre of kiEphB2/EphB2 cell clusters.

### 3.3. Role of N-cadherin

We find that N-cadherin is required both in EphB2 and ephrinB1 cells to promote segregation and enable border sharpening. Several lines of evidence support that N-cadherin enables homotypic cohesion that stabilizes cell organization. In segregation assays, knockdown of N-cadherin only in EphB2 cells leads to smaller EphB2 cell clusters that have fewer interconnections compared with control assays. Knockdown of N-cadherin leads to a major increase in homotypic repulsion, to a similar value as for heterotypic repulsion in the presence of N-cadherin. Time lapse movies reveal that the increased homotypic repulsion of EphB2 cells causes the forming clusters to break up, whereas in control EphB2/ephrinB1 assays the clusters are stable.

The results of simulations suggest that two aspects of cell behaviour contribute to the disruption of border sharpening by N-cadherin knockdown. First, the large increase in homotypic repulsion leads to a threefold decrease in the ratio of heterotypic compared with homotypic repulsion. In simulations using control EphB2 and ephrinB1 cell parameters, but with heterotypic repulsion reduced to the ratio that occurs after N-cadherin knockdown, there is a failure of border sharpening. Second, border sharpening is disrupted when there is a short duration of homotypic cell contact, which is only partly alleviated by increasing the relative strength of heterotypic repulsion. Furthermore, when parameters from N-cadherin knockdown are used in simulations, border sharpening is rescued when the duration of homotypic contact is increased. These findings suggest that the amount of homotypic cohesion and the ratio of heterotypic/homotypic repulsion each contribute to border sharpening, both of which are disrupted by N-cadherin knockdown.

Although initial studies emphasized that interacting Eph receptors and ephrins are in complementary domains [14], in many tissues this is accompanied by overlaps in expression. For example in early *Xenopus* embryos, mesoderm and ectoderm each express a combination of Eph receptors and ephrins such that high-affinity partners are in complementary tissues [16]. This creates bi-directional forward signalling that prevents mixing between these tissues, but also overlapping expression of lower-affinity partners [15,16]. The overlapping expression underlies homotypic repulsion that is counteracted by C-cadherin [16]. Our findings suggest that the homotypic repulsion of EphB2 cells is in part due to low-level endogenous expression of ephrinB1 and ephrinB2 in HEK293 cells. It is not known how homotypic repulsion is regulated in ephrinB1 cells, because knockdown of potential interacting Eph receptors decreases cell motility.

N-cadherin may counteract repulsion by mediating adhesion that needs to be overcome in order for cells to disengage, and/or by activating signalling pathways that antagonize Eph-mediated repulsion. Convergence of signalling may occur on Rho family GTPases, in which Eph-mediated activation of RhoA underlies cell repulsion [13,24], whereas cadherin clustering can activate Rac1 and increase actin assembly [37]. Indeed, recent studies suggest that signalling is the principal way that cadherins regulate cell adhesion strength [3]. It has been shown, for example, that in the pre-migratory neural crest, E-cadherin stabilizes adhesion by activating Rac at the cell–cell contact site [38]. N-cadherin has a distinct activity from E-cadherin, in which it mediates homotypic repulsion by polarizing Rac activity so that it is stronger distal from the cell–cell contact [38,39]. Thus, in the neural crest, N-cadherin promotes repulsion, whereas we find that, in HEK293 cells, it suppresses Eph–ephrin-mediated repulsion. It will be interesting to test whether the strong polarizing activity of Eph–ephrin signalling underlies this distinct relationship between N-cadherin and cell repulsion.

## 4. Material and methods

### 4.1. Cell culture and time lapse movies

Cells were cultured at 37°C with 5% CO_2_ in Dulbecco's modified Eagle's medium supplemented with 10% fetal calf serum, glutamine and antibiotics. Prior to an experiment, cells were labelled with CMFDA (green) or CMRA (red) cell tracker dyes (Molecular Probes, Invitrogen), and then dissociated with Accutase (Sigma).

For segregation assays, differently labelled cells were mixed in equal proportions, plated on a fibronectin-coated coverglass chambered slide (Lab-Tek) at a density of 200 000 cells cm^−2^ and cultured for 48 h before fixation. For cell tracking experiments, 20 000 labelled cells were placed into each well (0.7 cm^2^) of an eight-well chambered slide, and visualized using a Deltavision RT live-imaging workstation and Olympus IX-70 microscope with a 10×/0.4NA objective. Images were taken every 3 min for 16–22 h and were processed using ImageJ.

For boundary assays, a two-well culture insert (Ibidi) was placed onto a fibronectin-coated chambered slide (Lab-Tek) and 70 µl of labelled cells put into each side at a concentration of 1–1.26 million total cells ml^−1^ (0.22 cm^2^ growth area per well). Cells were incubated at 37°C for 6–12 h before the barrier was lifted and fresh medium added. Movies were captured as described above.

### 4.2. Quantitation of cell segregation and border sharpening

The size of EphB2 cell clusters was quantitated using particle analysis in ImageJ. Images were thresholded to remove noise, converted into binary data and then particle analysis applied, setting the minimal cluster size at 500 µm^2^. The area of clusters was converted into cell number based on a mean cell area of 200 µm^2^.

Boundary sharpness was quantitated by measuring the length of the boundary from greyscale images of one cell population based on a pixel intensity threshold. Boundary length was calculated from the sharpening simulations by searching for the nearest heterotypic neighbour for each cell, which gave a sequence of short segments between neighbouring cells. The line from the centre of each segment accurately represents the boundary between the two populations. The boundary length is calculated as the sum of the length of each of the lines between nearby centres.

### 4.3. Quantitation of cell behaviour

We analysed low-density cell assays to quantitate individual cell behaviour. For each experiment, cells were manually tracked using ImageJ for the duration of two movies (23 ± 1.8 cells per movie) and the contact events with another cell identified (27 ± 1.7 contacts per cell). For each contact, we measured the duration and determined if it ends with a collapse response. The duration of contact and the frequency of collapse (table 2) were directly derived from these data. We computed *θ*, the angle between the axis formed by the two cells at the beginning of contact (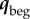) and the direction of the cell after contact (***v***_1_(*t*_end_)), where 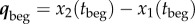 and 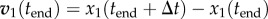 with Δ*t* = 3 frames. A cell for which *θ* is within the range *π*/2 to 3*π*/4 is considered to be migrating away from the other cell. The displacement during repulsion was calculated for cells that do not have a further collision within 20 min (21.4 ± 2.9 contact events).

**Table 2.** Mean duration of contact and frequency of repulsion.

	duration of contact (min)	frequency of repulsion
homotypic EphB2	72	0.47
heterotypic EphB2	24	0.84
homotypic kiEphB2	89	0.44
heterotypic kiEphB2	27	0.69
homotypic EphB2-siNcad	24	0.84
heterotypic EphB2-siNcad	15	0.94
homotypic ephrinB1	57	0.62
heterotypic ephrinB1	26	0.92
homotypic ephrinB1-siNcad	21	0.77
heterotypic ephrinB1-siNcad	15	0.81

### 4.4. Immunocytochemistry and western blotting

Cells were fixed for 15 min in 4% paraformaldehyde at 37°C, washed three times in PBS and stored at 4°C prior to immunostaining. The antibodies were anti-N-cadherin (BD Biosciences, 610920, 1 : 250) or anti-Pan-cadherin (Sigma, C-1821, 1 : 100). F-actin staining was carried out with 647N-Phalloidin (Sigma, 65906, 1 : 300). Cells were washed twice in PBT (0.1% Tween20 in PBS), blocked for 30 min and then incubated for 2–3 h with primary antibodies in 2.5% goat serum, 1% DMSO in PBT. After washing eight times in PBT during 1 h, cells were incubated for 2 h in secondary antibody (donkey anti-mouse Cy5 conjugated, Jackson ImmunoResearch, 1 : 400) together with DAPI nuclear counterstain. The cells were then washed eight times in PBT and mounted in FluorSave. Immunostained cells were imaged using a Zeiss LSM710 confocal microscope and images processed using ImageJ. Fiji was used to plot the intensity profile of cadherin and F-actin staining in boundary assays. Western blotting was carried out with N-cadherin (1 : 1000) or Pan-cadherin (1 : 500) antibodies.

### 4.5. Gene knockdown

siRNAs were from Dharmacon (Eph receptors, ephrins, N-cadherin: On-Target plus SMART pool) or Ambion (N-cadherin: Silence Select pre-designed). A non-targeting siRNA was used as negative control. Sixty picomoles of siRNA was transfected with Lipofectamine RNAiMAX (Invitrogen) according to the manufacturer's instructions. The transfected cells were incubated for 48 h before replating for assays.

### 4.6. Agent-based simulations

The simulations were based on those described previously [30,31] with modifications of the cell interactions to include adhesive, repulsive and cohesive behaviours. The code is deposited at https://github.com/anaiskhuong/SimCell. The model allows a choice of behaviour for each cell population: their interactions can be asymmetric, they can show different homotypic and heterotypic responses, and adhesion and repulsion parameters can be independently set. We chose a stochastic model in which probabilities are constant in time.

Each cell is modelled by its centroid and eight nodes which represent its discretized membrane. Cells are able to move, with the structure maintained by constraints on these nodes. Cell geometry is characterized by the radius that defines the most circular shape they can take, and an ideal distance between two adjacent nodes of their membrane (bonds), which prevents nodes from moving too far and limits cell spread. The membrane is deformable as each node can move independently because of its interactions with neighbouring cells, but these ideal bonds maintain the cell shape. Cells can neither overlap with each other nor escape the simulation area.

The node that is the leader gives the direction of cell migration, which in free migration is randomly chosen. The frequency of changing direction *η*_dir_ determines the persistence of migration. We modelled cell interactions by allowing nodes to create a link. The duration of cell contact is described with the rate of breaking a link between two cells *η*_adh_: the higher *η*_adh_ is, the quicker a link breaks. Homotypic and heterotypic adhesion can be assigned different rates. In cell–cell repulsion, when a link between two cells breaks, its opposite point is more likely to become a leader. The amount of repulsion is determined by the probability *p*(repulsion) of this biased choice of opposite direction. The model includes cohesive migration in which a repelled cell pulls on neighbours with which it has adhesive links, thus influencing their direction of migration.

### 4.7. Parameters used in simulations

Each cell has a diameter of 15 µm, with a velocity of 0.75 µm per min and *p*(change of direction) of 0.2. Eight hundred cells were simulated in an area of diameter 500 µm. One time step corresponds to 0.5 s, and thus 50 000 time steps in the simulation corresponds to approximately 7 h, consistent with the experimental time scale. We estimated the adhesion and repulsion parameters from the quantitations of the duration and frequency of repulsion (table 2). *p*(repulsion) is the frequency of repulsion. The rate for a link to break is the inverse of the duration of contact, and the probability to break a link *p*(break) = 1−exp(−*η*_adh_). The calculated values of *p*(break) are given in table 3.

**Table 3.** *p*(break) values used in simulations.

	EphB2	ephrinB2	kiEphB2
homotypic control	0.014	0.018	0.011
heterotypic control	0.041	0.038	0.036
homotypic Ncad KD	0.041	0.046	
heterotypic Ncad KD	0.063	0.063	

## Supplementary Material

Captions for movies and Suppl. Figs

## Supplementary Material

Suppl. Fig. 1. Quantitation of cadherin, F-actin and border shifting.

## Supplementary Material

Suppl. Fig. 2. Direction of cell migration after heterotypic and homotypic contact.

## Supplementary Material

Suppl. Fig. 3. Time course of simulations of cell segregation.

## Supplementary Material

Suppl. Fig. 4. Time course of simulations of border sharpening.

## Data accessibility

Datasets and movies supporting this article have been uploaded as part of the electronic supplementary material. The code for the simulations is deposited at https://github.com/anaiskhuong/SimCell.
