# Supplementary material for: Cell segregation and border sharpening by Eph receptor–ephrin-mediated heterotypic repulsion
Source: J R Soc Interface. 2017 Jul 26;14(132):20170338. doi: 10.1098/rsif.2017.0338 (PMC5550979; doi:10.1098/rsif.2017.0338)
Supplement: Captions for movies and Suppl. Figs [file rsif20170338supp1.doc]

**Captions for movies and Suppl. Figs**

**MOVIES**

Excerpts from time lapse movies that illustrate cell behaviour and segregation. All movies have a frame interval of 3 min.

**Movie 1:** EphB2 cells (green) and ephrinB1 cells (red) at high density during late stages of segregation. Both cell populations are motile and exhibit homotypic and heterotypic repulsion responses. EphB2 cell clusters become increasingly compacted.

**Movie 2:** Repulsion responses following interactions between EphB2 cells (green) and ephrinB1 cells (red) at low density.

**Movie 3:** Repulsion responses following interactions between kiEphB2 cells (green) and ephrinB1 cells (red) at low density.

**Movie 4:** Homotypic repulsion responses following interactions between EphB2 cells (green) at low density.

**Movie 5:** Homotypic repulsion responses following interactions between kiEphB2 cells (green) at low density.

**Movie 6:** Homotypic repulsion responses following interactions between ephrinB1 cells (green) at low density.

**Movie 7:** Early stages of cell segregation in low density culture of EphB2 cells (green) and ephrinB1 cells (red). Repeated heterotypic repulsion events are seen to push EphB2 cells together into close contact in a cluster, which is maintained despite homotypic repulsion.

**Movie 8**: Early stages of cell segregation in low density culture of EphB2 cells (green) and ephrinB1 cells (red) following knockdown of N-cadherin. Although a cluster of EphB2 cells has formed, it is broken up by strong homotypic repulsion.

**SUPPLEMENTAL FIGURES**

**Suppl. Fig. 1. Quantitation of cadherin, F-actin and border shifting.** Images of N-cadherin (A, C) and F-actin (B, D) staining in EphB2-ephrinB1 boundary assays (see Fig.2D, E) were analysed to compare the intensity of signal at heterotypic (blue arrow) and homotypic (green and red arrows) contacts. (A, B): Where EphB2 and ephrinB1 cells are in contact, there is no reduction in N-cadherin staining compared with EphB2 or ephrinB1 homotypic contacts. F-actin staining is not specifically localised at the heterotypic border. (C, D): Where there is a gap between EphB2 and ephrinB1 cells, strong F-actin staining is detected in cells at the free edge (blue arrows) in association with retraction fibres (purple arrows) suggestive of repulsion. N-cadherin levels are lower at the free edge than at homotypic EphB2 or ephrinB1 contacts, except at some locations of strong F-actin staining.

**Suppl. Fig. 2. Direction of cell migration after heterotypic and homotypic contact.** The plots show the direction of cell movement after cell-cell contact, in which 0 corresponds to no change in direction. The length of each segment indicates the number of contact events. These data were used to calculate the proportion of cells that move away after contact shown in Fig.3J.

**Suppl. Fig. 3. Time course of simulations of cell segregation.** Snapshots at 10K step intervals of the simulations of cell segregation shown in Fig. 4.

(A) Directional repulsion and adhesion (contact duration) values of EphB2 and ephrinB1 cells; (B) Directional repulsion and adhesion values of kiEphB2 and ephrinB1 cells; (C) Adhesion values of EphB2 and ephrinB1 cells with random migration; (D) 500:1 ratio of homotypic:heterotypic contact duration with random migration.

**Suppl. Fig. 4. Time course of simulations of border sharpening.** Snapshots at 10K step intervals of the simulations of border sharpening shown in Fig. 4.

(A) Directional repulsion and adhesion values of EphB2 and ephrinB1 cells; (B) Directional repulsion and adhesion values of kiEphB2 and ephrinB1 cells; (C) Adhesion values of EphB2 and ephrinB1 cells with random migration; (D) 500:1 ratio of homotypic:heterotypic contact duration with random migration.
