# Supplementary figures and images for: Cell segregation and border sharpening by Eph receptor–ephrin-mediated heterotypic repulsion
Source: J R Soc Interface. 2017 Jul 26;14(132):20170338. doi: 10.1098/rsif.2017.0338 (PMC5550979; doi:10.1098/rsif.2017.0338)

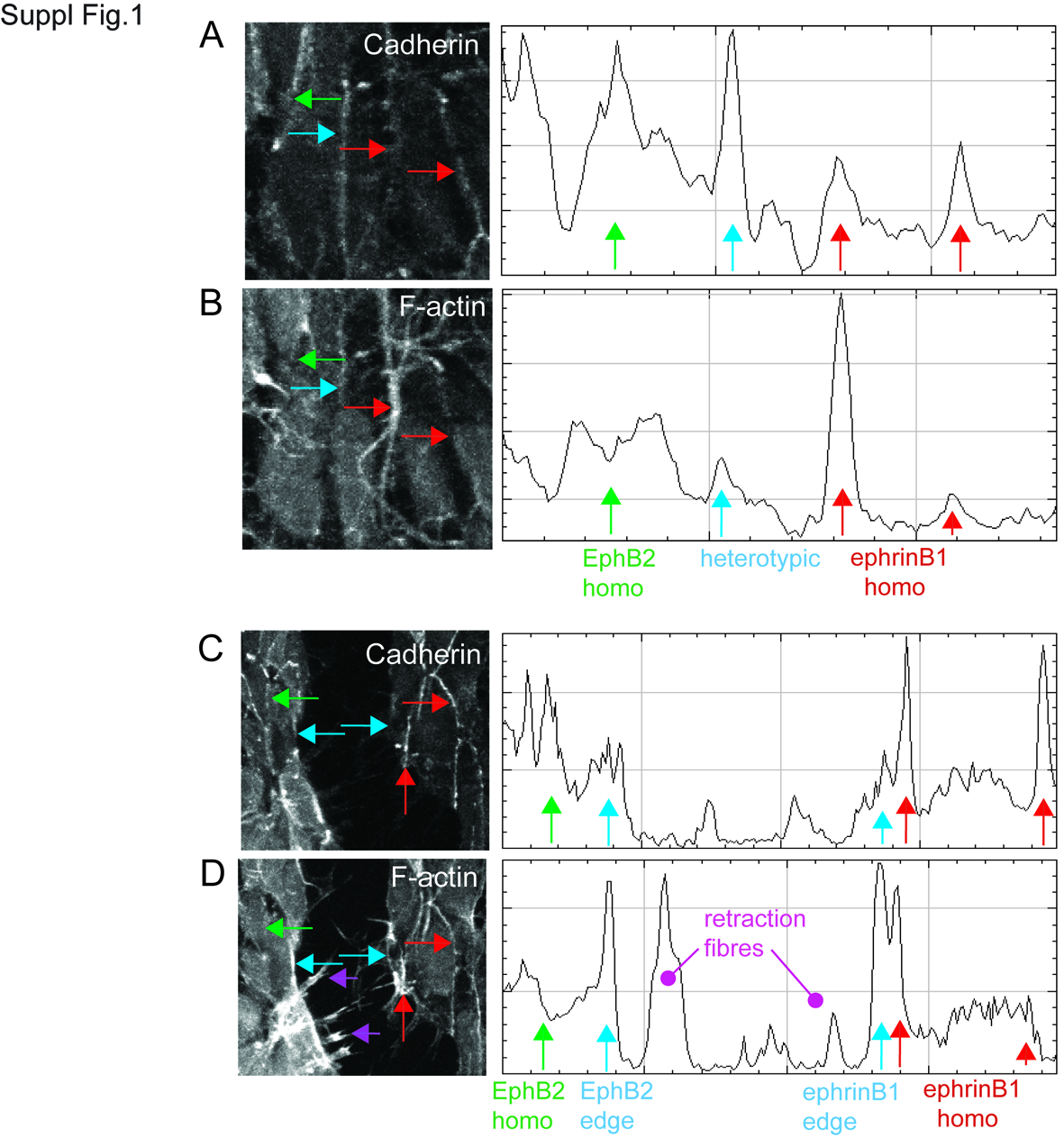

Supplement: Suppl. Fig. 1. Quantitation of cadherin, F-actin and border shifting. [file rsif20170338supp2.tif]

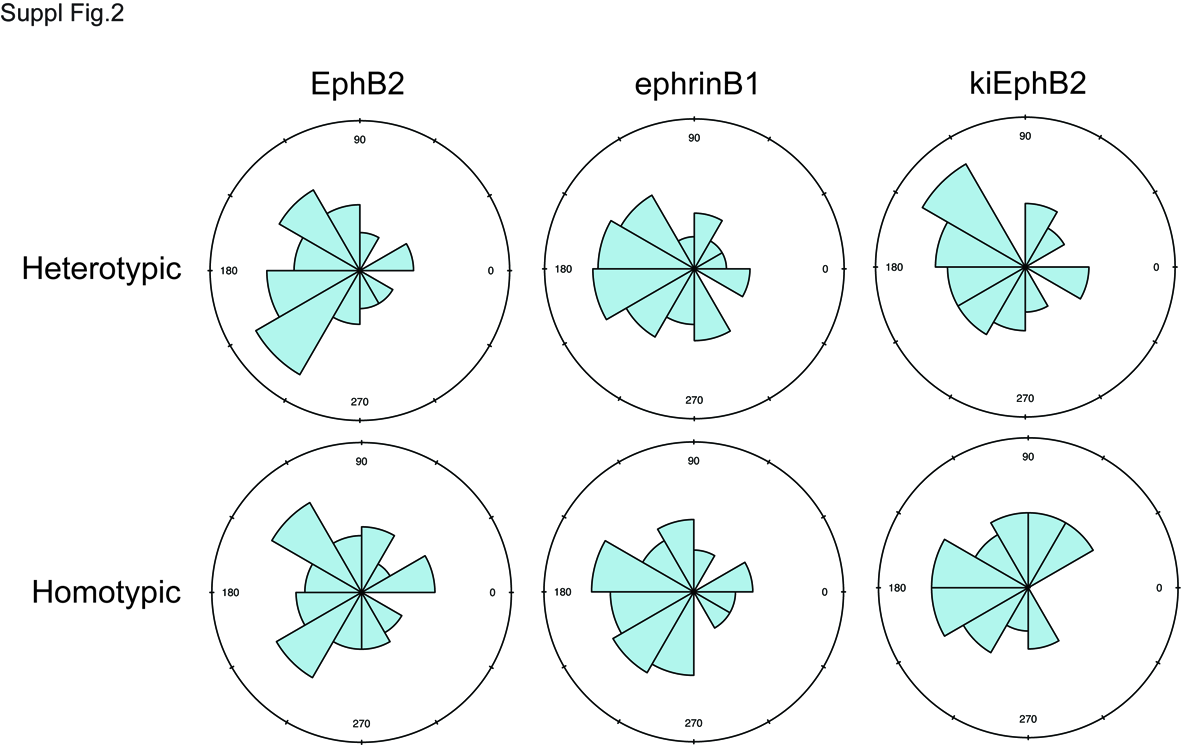

Supplement: Suppl. Fig. 2. Direction of cell migration after heterotypic and homotypic contact. [file rsif20170338supp3.tif]

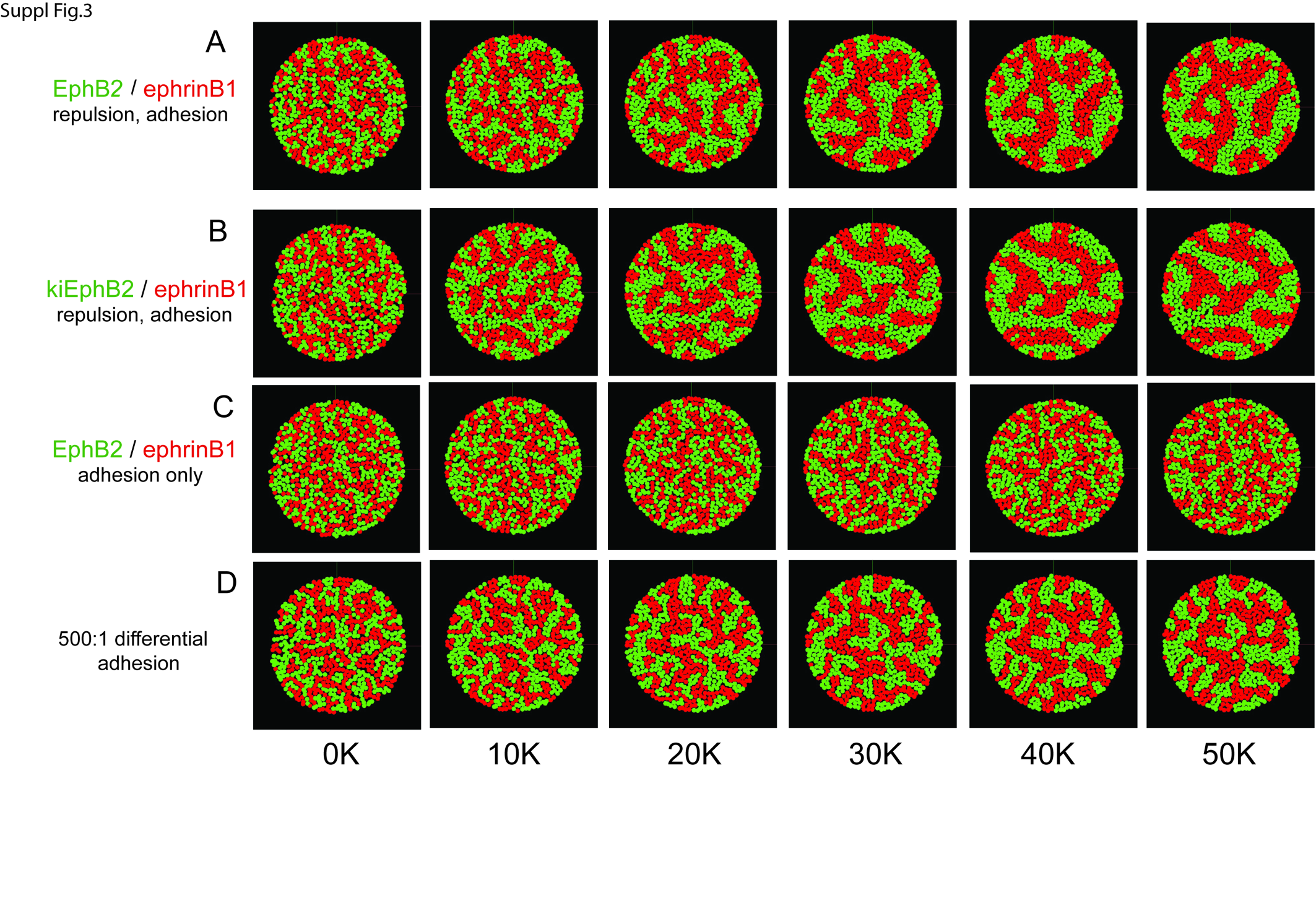

Supplement: Suppl. Fig. 3. Time course of simulations of cell segregation. [file rsif20170338supp4.tif]

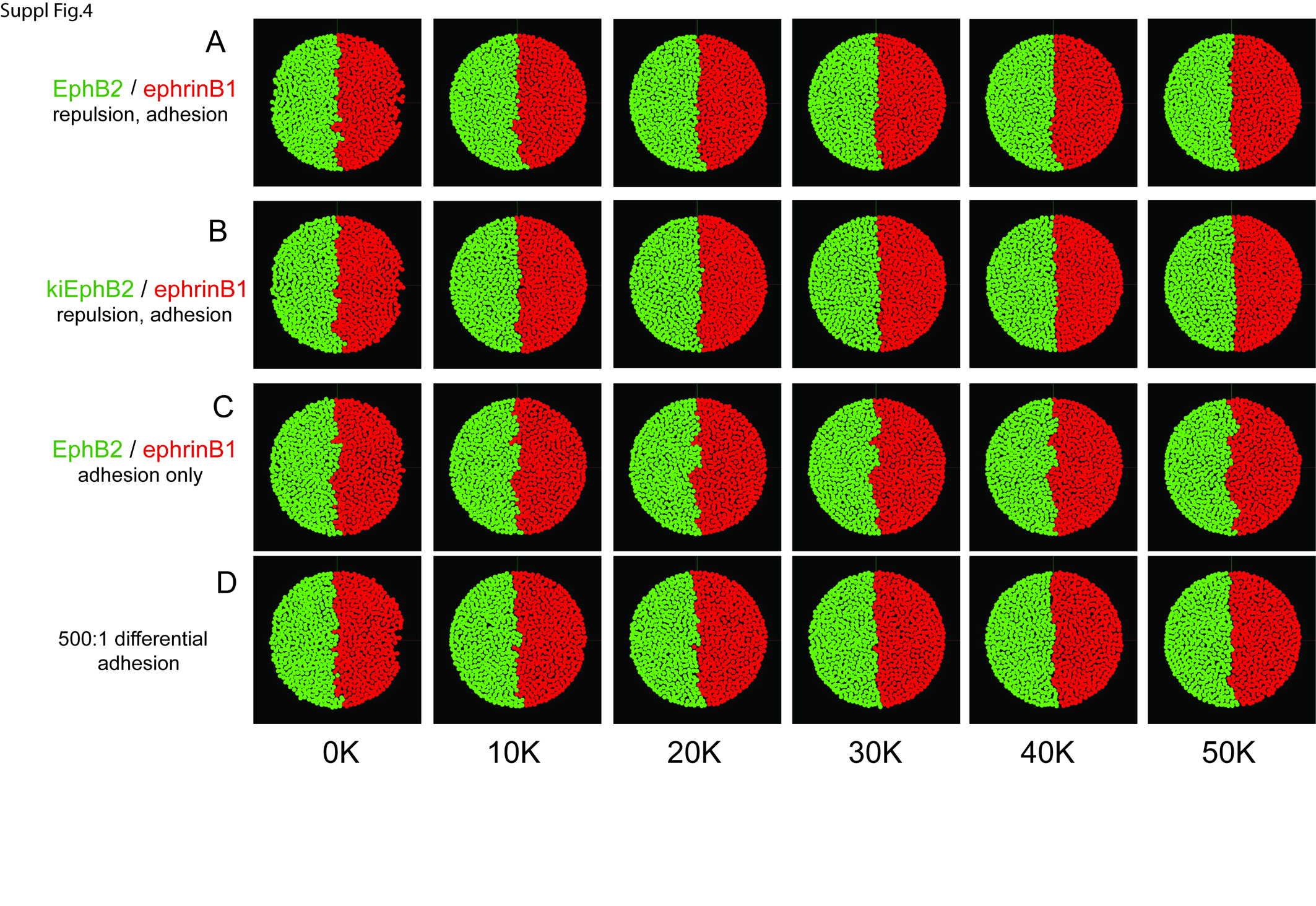

Supplement: Suppl. Fig. 4. Time course of simulations of border sharpening. [file rsif20170338supp5.tif]
